# War Memorial Hospital in Zululand

**Published:** 1921-04-30

**Authors:** 


### War Memorial Hospital in Zululand.

Zululand until not long ago was inhabited by naked savages. But the coastlands, of which Empangeni may be said to be the capital, are now largely under sugar cultivation by Natal colonists, and the country is prospering.

Empangeni was *en fête* on March 3, when his Honour the Administrator formally opened the hospital which has been erected by the residents of the district as their war memorial. The hospital is known as the Lower Umfolosi District War Memorial Hospital. The building has cost £6,000, and the furniture a little over £1,000. The various sugar-milling companies have given generously. The memorial is one which redounds to the foresight of the little British community. The Chairman of the Hospital Committee is Lieut.-Colonel R. M. Tanner, D.S.O., the resident magistrate, brother of General Tanner, who latterly commanded the South African Brigade in France. Amongst those who were also present at the opening were Dr. J. H. Balfe, Medical Superintendent of Natal Government Hospitals, and Dr. G. K. Moberley. A beautiful brass was unveiled by the Administrator, engraved with the names of local heroes fallen in the war.

Part of the hospital has been used since September last, and fifty-six patients admitted. The nursing staff consisted of the Matron, Miss Ingle, Sisters Van Niekerk and Flower, and Probationer Violet Gibney.

